# Active-site copper reduction promotes substrate binding of fungal lytic polysaccharide monooxygenase and reduces stability

**DOI:** 10.1074/jbc.RA117.000109

**Published:** 2017-12-19

**Authors:** Daniel Kracher, Martina Andlar, Paul G. Furtmüller, Roland Ludwig

**Affiliations:** From the ‡ Biocatalysis and Biosensing Research Group, Department of Food Science and Technology, and; the § Division of Biochemistry, Department of Chemistry, BOKU–University of Natural Resources and Life Sciences, Muthgasse 18, 1190 Vienna, Austria

**Keywords:** metalloenzyme, protein stability, carbohydrate-binding protein, plant cell wall, polysaccharide, Active-site copper, lytic polysaccharide monooxygenase

## Abstract

Lytic polysaccharide monooxygenases (LPMOs) are a class of copper-containing enzymes that oxidatively degrade insoluble plant polysaccharides and soluble oligosaccharides. Upon reductive activation, they cleave the substrate and promote biomass degradation by hydrolytic enzymes. In this study, we employed LPMO9C from *Neurospora crassa*, which is active toward cellulose and soluble β-glucans, to study the enzyme-substrate interaction and thermal stability. Binding studies showed that the reduction of the mononuclear active-site copper by ascorbic acid increased the affinity and the maximum binding capacity of LPMO for cellulose. The reduced redox state of the active-site copper and not the subsequent formation of the activated oxygen species increased the affinity toward cellulose. The lower affinity of oxidized LPMO could support its desorption after catalysis and allow hydrolases to access the cleavage site. It also suggests that the copper reduction is not necessarily performed in the substrate-bound state of LPMO. Differential scanning fluorimetry showed a stabilizing effect of the substrates cellulose and xyloglucan on the apparent transition midpoint temperature of the reduced, catalytically active enzyme. Oxidative auto-inactivation and destabilization were observed in the absence of a suitable substrate. Our data reveal the determinants of LPMO stability under turnover and non-turnover conditions and indicate that the reduction of the active-site copper initiates substrate binding.

## Introduction

Fungal cellulose degradation depends on a set of *endo-* and *exo*-acting glycosyl hydrolases that cleave the glucan chains in cellulose into soluble cellobiose moieties (1). In 2010, an auxiliary enzyme was identified that employs a redox mechanism to cleave glycosidic bonds (2–5). Fungal copper–dependent LPMOs^4^ (CAZy family AA9) cleave polymeric substrates, including crystalline cellulose and hemicelluloses (6–9). Whereas substrate degradation by LPMO alone is not very efficient, it exerts a boosting effect on associated cellulases (4, 10). All known LPMOs share two strictly conserved histidine residues in a constellation termed the “histidine brace” to coordinate the active-site copper atom (5). To act on a planar substrate like cellulose, the solvent-exposed active site is located on a flat “binding face,” which is oriented toward the substrate surface during catalysis (11, 12). Thus, LPMOs do not depend on the accessibility of single glucan chains. Analysis of reaction products revealed that LPMOs hydroxylate the C4 or C1 carbons of glucosyl moieties adjacent to the glyosidic bond (13, 14), leading to strand breaks, which are starting points for hydrolytic enzymes (15). The detailed catalytic mechanism by which LPMOs facilitate substrate cleavage still needs experimental clarification. It is generally accepted that the first reaction step of LPMO is the reduction of the active-site copper by small molecule reductants (16, 17) or by the fungal flavocytochrome cellobiose dehydrogenase (3, 4, 17). In the second step, the recruitment of molecular oxygen as cosubstrate and the formation of a copper-bound superoxide or oxyl intermediate has been proposed (13, 18–20). The timing of the delivery of the second electron to the reaction differs according to the suggested reactive oxygen species (21). Recently, hydrogen peroxide was reported as cosubstrate, resulting in higher turnover rates than obtained with oxygen (22, 23). The crystal structure of a fungal LPMO in complex with an oligosaccharide provided valuable structural insights into the substrate targeting of LPMOs (24). Substrate binding and regioselectivity of LPMO arise from interactions of aromatic and hydrophilic amino acids located on distinct loops flanking the active site, which position the copper center onto the substrate (12, 25, 26). In a recent AFM study, Eibinger *et al.* (27) found that LPMO in the presence of the reductant ascorbic acid is bound on the substrate surface for a considerable time and shows a low dissociation rate constant of 0.97 min^−1^. Experimental evidence suggests that copper reduction, cosubstrate activation, and interaction with carbohydrate substrates occur in the vicinity of the copper center (26, 28), which requires well-timed, sequential reaction steps. Due to the mostly insoluble nature of the substrates, the application of standard biochemical methods to examine catalytic and molecular properties of LPMOs is challenging. In the present work, we used a fluorimetric assay in combination with spectroscopic methods to investigate the thermal stability of a fungal LPMO in solution and during its interaction with carbohydrate substrates. To date, only limited information on the thermal stability of LPMOs is available. An analysis of four *Neurospora crassa* LPMOs in solution using differential scanning calorimetry revealed transition midpoint temperatures ranging from 63 to 68 °C (29). Using differential scanning fluorimetry, an apparent transition midpoint temperature (*T_m_*) value of 65 °C was measured for the copper-saturated bacterial LPMO from *Bacillus amyloliquefaciens* (30). In a first attempt to increase thermal stability by enzyme engineering, additional disulfide bridges were introduced into *Streptomyces coelicolor* LPMO, which increased the *T_m_* value of the enzyme from 51 to 63 °C (31).

In this study, we employed LPMO9C from the ascomycete *N. crassa*, consisting of a catalytic domain connected to a family 1 carbohydrate-binding module (CBM1) via a linker peptide of 82 amino acids. This enzyme was shown to cleave cellulose as well as several soluble hemicelluloses and soluble cello-oligomers (7, 14, 32) and is therefore accessible by several biochemical methods. *Nc*LPMO9C's broad substrate spectrum allowed us to explore key biophysical parameters in solution, which are of biological and technological relevance.

## Results

### Thermal stability of NcLPMO9C

**Figure 1.**
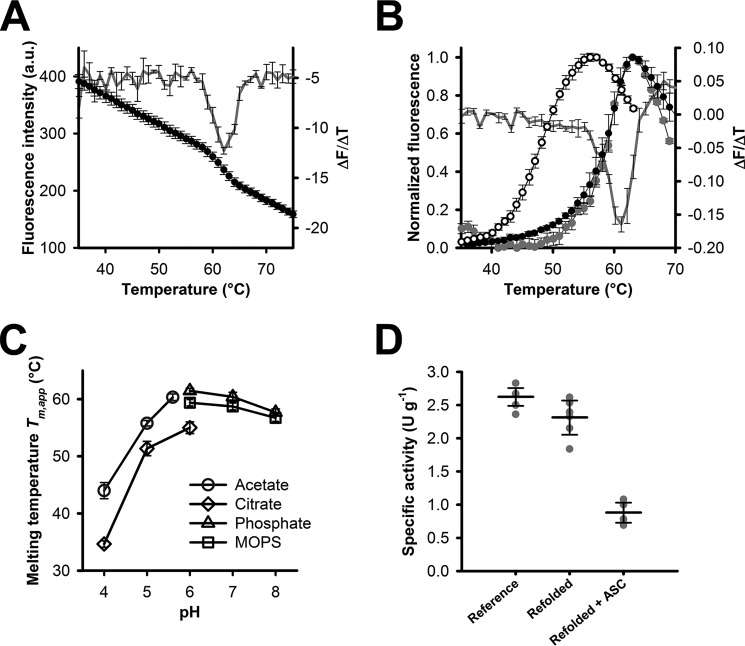
**Fluorimetric determination of *T_m_*_,app_ values of *Nc*LPMO9C and activity measurements**. *A*, determination of the *T_m_*_,app_ of *Nc*LPMO9C (10 μm) on the basis of the intrinsic tryptophan fluorescence. Unfolding was measured at a rate of 1 °C min^−1^ (*black circles*) in 50 mm phosphate buffer, pH 6.0, by recording the emission at 349 nm upon excitation at 279 nm. The first derivative of the fluorescence traces is shown as a *gray curve. B*, determination of *T_m_*_,app_ via measurement of the ANS-protein complex formation (λ_ex_ = 378 nm; λ_em_ = 480 nm) for oxidized LPMO (*filled circles*) and LPMO incubated with 5 mm ascorbic acid (*empty circles*). Data were recorded in 50 mm phosphate buffer, pH 6.0, at an LPMO concentration of 5 μm and a scan rate of 1 °C min^−1^. Refolding of oxidized LPMO was measured under the same conditions by decreasing the temperature from 70 to 30 °C in steps of 1 °C min^−1^ (*gray circles*). The *gray line* shows the first derivative of fluorescence traces of the unfolding reaction. *C*, pH– and buffer species–dependent *T_m_*_,app_ values of LPMO (5 μm) measured with ANS (200 μm) using the same experimental setup as in *B* and a molarity of 50 mm for all buffer salts. *D*, activity of refolded LPMO was measured based on the futile peroxide-producing activity pf LPMO in the absence of substrate. LPMO (5 μm) in the absence or presence of ascorbic acid (5 mm) was subjected to a heat ramp from 30 to 75 °C, followed by a decrease from 70 to 30 °C at a scan speed of 1 °C min^−1^. ANS was excluded in these experiments to avoid interference with the activity assay. After the heat treatment, LPMO samples were rebuffered using concentration tubes with a cut-off of 10 kDa until the flow-through was free of ascorbic acid (determined photometrically at 270 nm) (*Refolded* + *ASC*). The same procedure was applied to an LPMO control without heat treatment (*Reference*) and to refolded, oxidized LPMO (*Refolded*). Twenty μl of concentrated LPMO solutions with protein concentrations ranging from 0.3 to 0.5 mg ml^−1^ were used for the Amplex Red/horseradish peroxidase assay as described under “Experimental procedures.” All data are expressed as mean values ± S.D. (*error bars*) from three (*A*, *B*, and *C*) or six measurements (*D*).

We used a fluorescence-based unfolding assay to analyze the stability of *Nc*LPMO9C. Initial attempts to measure thermal unfolding via LPMO's intrinsic protein fluorescence showed only a minor intensity difference between the folded and the unfolded state, even at a high protein concentration of 10 μm (Fig. 1*A*). *Nc*LPMO9C, without its CBM1 and its protein linker, contains four tryptophan residues; however, their location is close to the protein surface (32), which reduced the quantum yield (Fig. S1, *A* and *B*). The addition of the hydrophobic dye 8-anilinonaphthalene-1-sulfonic acid (ANS) resulted in distinct fluorescence signals upon protein unfolding and allowed us to determine a defined transition point (Fig. 1*B* and Fig. S2). From these experiments, apparent midpoint transition temperatures, hereafter referred to as *T_m_*_,app_, were determined from the first derivative of the fluorescence traces (Fig. 1*B*). For the oxidized enzyme, a *T_m_*_,app_ of 61.5 ± 0.4 °C was measured, which was in good agreement with the *T_m_*_,app_ approximated from the low intrinsic protein fluorescence (62.1 ± 0.1 °C). Both values correspond well to the published midpoint transition temperature of *Nc*LPMO9C (63.0 ± 0.1 °C) determined by differential scanning calorimetry under the same experimental conditions (29). Consequently, unfolding in the presence of ANS was used to determine the *T_m_*_,app_ of *Nc*LPMO9C for different conditions. Unfolding data revealed pronounced effects of the buffer system and the pH milieu on the thermal stability of *Nc*LPMO9C (Fig. 1*C*). *T_m_*_,app_ values at and above 60 °C were found for buffers between pH 6 and pH 8, but *T_m_*_,app_ decreased in more acidic conditions. Compared with the highest *T_m_*_,app_ measured at pH 6.0 in phosphate buffer, the *T_m_*_,app_ in acetate buffer, pH 4.0, decreased by 17.5 °C, and the value in citrate buffer, pH 4.0, decreased by 26.8 °C. Hence, all of the following experiments were performed in 50 mm phosphate buffer at a pH of 6.0 unless stated otherwise. After complete unfolding at 75 °C and at a pH of 6.0, no visible precipitations occurred. When reducing the temperature from 70 to 30 °C at a rate of 1 °C min^−1^, a reversible time trace with a similar overall shape and a *T_m_*_,app_ of 60.5 ± 0.5 °C was observed, suggesting conditions of equilibrium unfolding (Fig. 1*B*, *gray circles*). The refolded enzyme retained ∼90% of its oxygen-reducing side activity (Fig. 1*D*), an uncoupling reaction previously reported and used to measure LPMO activity (29).

### Reduction of the active-site copper reduces stability

The addition of ascorbic acid in millimolar concentrations completely quenched the intrinsic tryptophan fluorescence (Fig. S1*B*). However, ANS as fluorescence probe showed stable fluorescence intensities in the presence of ascorbic acid. The emission intensities increased ∼1.8-fold when compared with the oxidized enzyme (Fig. S3, *A* and *B*). Under reducing conditions, the *T_m_*_,app_ of LPMO decreased by 12.7 °C (Table 1 and Fig. 1*B* (*empty circles*)). Measurement of the oxygen-reducing activity of LPMO incubated with ascorbic acid after reverting the temperature ramp from 70 to 30 °C at a rate of 1 °C min^−1^ showed that the enzyme retained only ∼33% of its initial activity (Fig. 1*D*). This is considerably lower than observed for the refolding of the oxidized enzyme (∼90%) and corroborates the previous finding that reducing conditions exert a destabilizing effect on the LPMO structure (22).

**Table 1:** ***T_m_*_,app_ of *Nc*LPMO9C in the presence of various reducing agents** Reducing agents were added to a final concentration of 2 mm, and EDTA was added to a final concentration of 300 μm. *T_m_*_,app_ values were measured in the presence of ANS, as outlined under “Experimental procedures.” Data are expressed as mean values ± S.D. from three independent repeats.

Additive	Without substrate	Xyloglucan (2 mg ml^−1^)
None	61.5 ± 0.4	61.4 ± 0.6
Ascorbic acid	48.8 ± 1.1	60.4 ± 0.5
Pyrogallol	50.6 ± 0.9	62.0 ± 0.1
Methoxyhydroquinone	49.6 ± 0.4	62.0 ± 0.8
Gallic acid	57.1 ± 0.1	58.4 ± 0.5
EDTA	52.7 ± 0.6	53.0 ± 1.0

In addition to ascorbic acid, we also tested other reducing agents that were previously shown to activate LPMO (17). At a concentration of 2 mm, all of these reductants decreased the thermal stability, albeit to varying degrees (Table 1 and Fig. S4, *A* and *B*). Gallic acid had the least effect on the thermal stability and decreased the *T_m_*_,app_ of the LPMO by 4 °C. It has to be noted that pyrogallol and methoxybenzenediol quenched the fluorescence signal of ANS ∼4-fold. The addition of the substrate xyloglucan recovered the stability of the LPMO. For methoxybenzenediol, ascorbic acid, and pyrogallol, the *T_m_*_,app_ in the presence of xyloglucan increased by 12.4, 11.6, and 11.4 °C, respectively. In the case of gallic acid, xyloglucan recovered *T_m_*_,app_ by ∼1.3 °C (Table 1 and Fig. S4 (*A* and *B*)).

To verify that the observed stabilizing effects originated from substrate interaction (binding or catalytic turnover) rather than from nonspecific stabilization effects, we tested polysaccharides that do not contain lengthy β-glucan structures and hence are no substrates for LPMO. The α-1,6–linked and α-1,3–branched dextran did not stabilize reduced LPMO. This was also observed for partially soluble xylan from birchwood, arabinogalactan, starch-derived maltodextrin, and cellobiose (Fig. S4*C*). Fluorescence emission traces obtained in the presence of these polysaccharides do not differ from traces recorded either for oxidized LPMO or reduced LPMO.

### The addition of carbohydrate substrates recovers the stability of LPMO in presence of reductants

Fluorescence traces recorded in the presence of ascorbic acid showed a much earlier onset of unfolding and a notably broader transition range when compared with the oxidized enzyme (Fig. 1*B*). After completion of the unfolding assays, a minimum concentration of 4.2 mm of ascorbic acid was still present (Fig. S3*C*), showing that reducing conditions were maintained throughout the unfolding assays.

**Figure 2.**
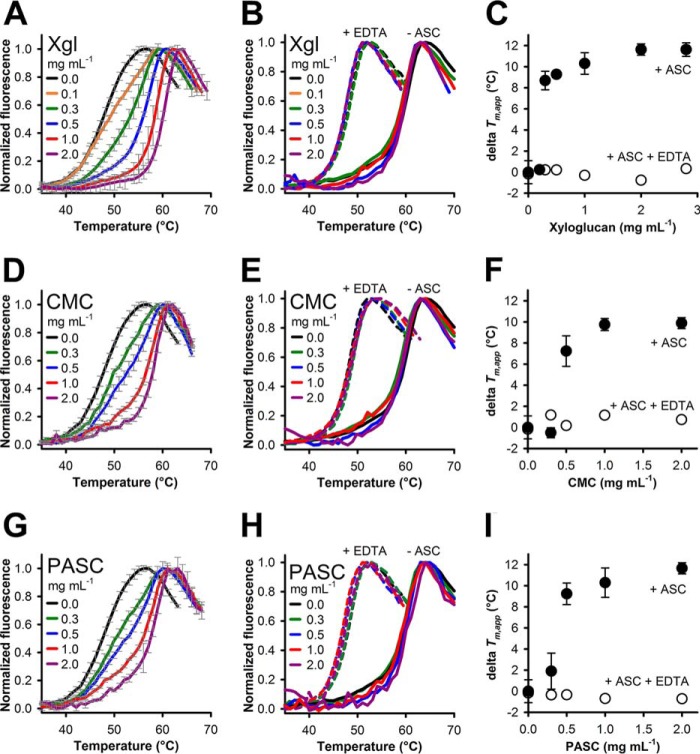
**Stability of oxidized and reduced LPMO in the presence of carbohydrate substrates and EDTA.** Stability of LPMO (5 μm) was measured with ANS (200 μm) in the presence of ASC (5 mm) and increasing concentrations of xyloglucan (*Xgl*; 0–2.8 mg ml^−1^; *A*), CMC (0–2 mg ml^−1^; *D*), or PASC (0–2 mg ml^−1^; *G*). The same reactions were carried out in the presence of 300 μm EDTA (*B*, *E*, and *H*, *dashed lines*) or in the absence of ascorbic acid and EDTA (*B*, *E*, and *H*, *solid lines*). The difference in *T_m_*_,app_ between reactions with ascorbic acid and ascorbic acid + EDTA (Δ*T_m_*_,app_) plotted against the substrate concentration is shown in *C* (xyloglucan), *F* (CMC), and *I* (PASC). All data were measured under aerobic conditions (oxygen concentration ∼240 μm) in 50 mm sodium phosphate buffer, pH 6.0, by measuring the emission at 480 nm upon excitation at 378 nm. Data are expressed as mean values ± S.D. (*error bars*) from three measurements (*A*, *D*, and *G*) or shown as average traces from two independent measurements (*B*, *E*, and *H*; experimental error within 5%).

The following experiments tested the effect of substrates on the thermal stability of LPMO. The substrate spectrum of *Nc*LPMO9C comprises cellulosic substrates and soluble hemicellulosic polymers, which were added to the unfolding experiments at increasing concentrations. Unfolding assays performed in the presence of ascorbic acid in combination with xyloglucan (*Xgl*; Fig. 2*A*), carboxymethylcellulose (CMC) (Fig. 2*D*), or phosphoric acid-swollen cellulose (PASC) (Fig. 2*G*) showed a concentration-dependent shift of the fluorescence emission traces toward higher temperatures. For all substrates, the transitions from the folded to the unfolded states gradually sharpened with increasing substrate concentrations. The calculated *T_m_*_,app_ values of *Nc*LPMO9C at the highest employed substrate concentrations were close to or above 60 °C, which is comparable with the *T_m_*_,app_ of the oxidized enzyme. Table 2 summarizes the stability data from Fig. 2.

**Table 2:**
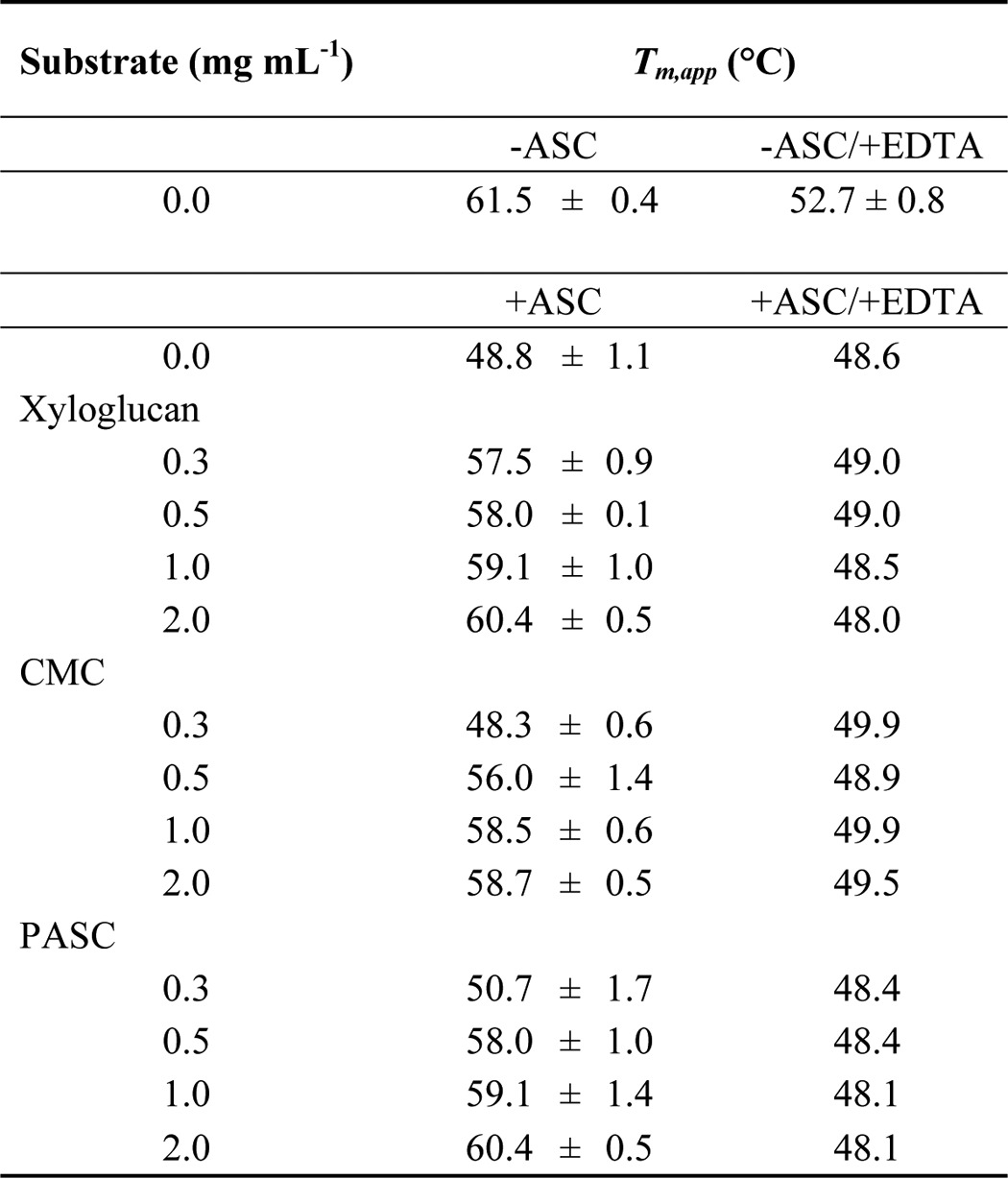
**Effect of substrate concentration, ascorbic acid, and EDTA on the thermal stability of *Nc*LPMO9C** *T_m_*_,app_ values were derived from the minimum of the first derivative of the melting curves. For stability measurements in presence of ascorbic acid (5 mm), the average ± S.D. of three independent repeats is given. For measurements with EDTA (300 μm), the mean of two repeats is given. The maximum deviation between these measurements was 0.5 °C.

Control experiments in which the metal chelator EDTA was added to otherwise identical unfolding assays led to a general loss of thermal stability (Fig. 2, *B*, *E*, and *H*, *dashed lines*). It is well-documented that EDTA removes the copper atom from the LPMO active site (*e.g.* see Ref. 2 and this study). For all investigated substrates and substrate concentrations, unfolding traces in the presence of EDTA were superimposable, and an overall *T_m_*_,app_ of 48.8 ± 0.6 was calculated (Table 2). This is identical to the *T_m_*_,app_ of EDTA-treated LPMO in the presence of ascorbic acid without any substrate (48.6 ± 0.6 °C). The loss of substrate stabilization observed in these experiments demonstrates the importance of the active-site copper for substrate interaction. When ascorbic acid was omitted in the unfolding assays, the addition of xyloglucan, CMC, or PASC did not increase the *T_m_*_,app_ of oxidized LPMO, suggesting the loss of structural integrity above ∼61 °C (Fig. 2, *B*, *E*, and *H*, *solid lines*).

**Figure 3.**
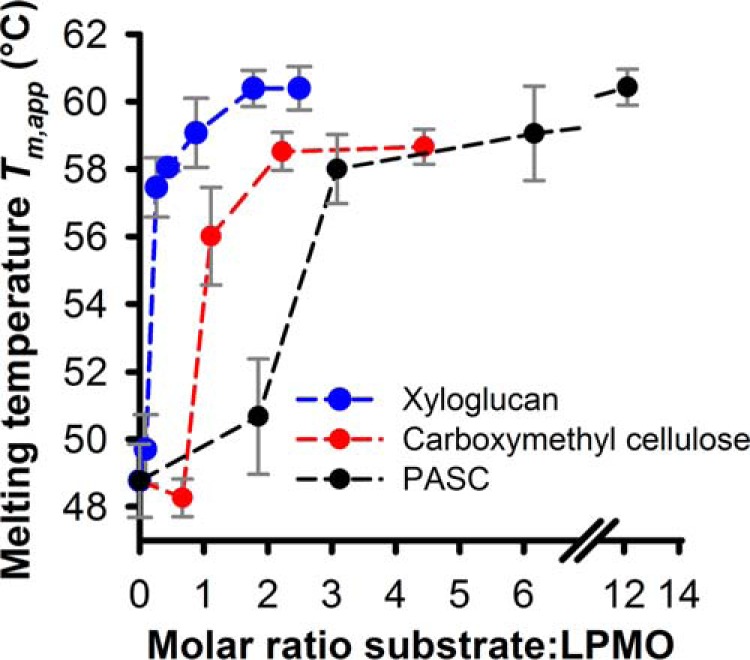
***T_m_*_,app_ values of LPMO plotted against the molar ratios of substrate/LPMO.** Data were taken from Fig. 2, *C*, *F*, and *I*. The molar concentration of the substrate repeats was calculated based on the approximate molecular masses of the polysaccharides (see “Results” for details).

These unfolding experiments were carried out under turnover conditions, in which substrate cleavage occurred until the thermal denaturation of LPMO. At low substrate concentrations, the substrate depletion over the course of the experiments (∼40 min) may limit the availability of LPMO-binding sites. The increase in stability depended on the substrate concentration, which is indicated by a shift of fluorescence traces. Substrate concentrations < 0.3 mg ml^−1^ had little impact on the thermal stability, but a steep jump of the *T_m_*_,app_ values was observed at higher concentrations (Fig. 2, *C*, *F*, and *I*). This suggests that the substrate concentration has to be high enough to saturate the active site/binding site to exert a stabilizing effect. An approximate concentration of oligomeric repeats present in the experiment was calculated based on the molecular masses of the substrates. The approximate molecular mass of xyloglucan from *Tamarind* is 225 kDa (32), and the molecular mass of PASC is 32.4 kDa based on the estimated chain length of 200 (33). The average molecular mass of CMC is 90 kDa (data from supplier). Fig. 3 plots *T_m_*_,app_ values of *Nc*LPMO9C *versus* the calculated molar ratio of oligomeric repeats/LPMO. In all cases, saturation was achieved at low molar ratios of ∼2:1 (xyloglucan and CMC) or 3:1 (PASC). It has to be noted that these values are an approximation, because the binding stoichiometry of LPMO for these substrates is not known with certainty (32).

### Effect of reactive oxygen species on LPMO stability

**Figure 4.**
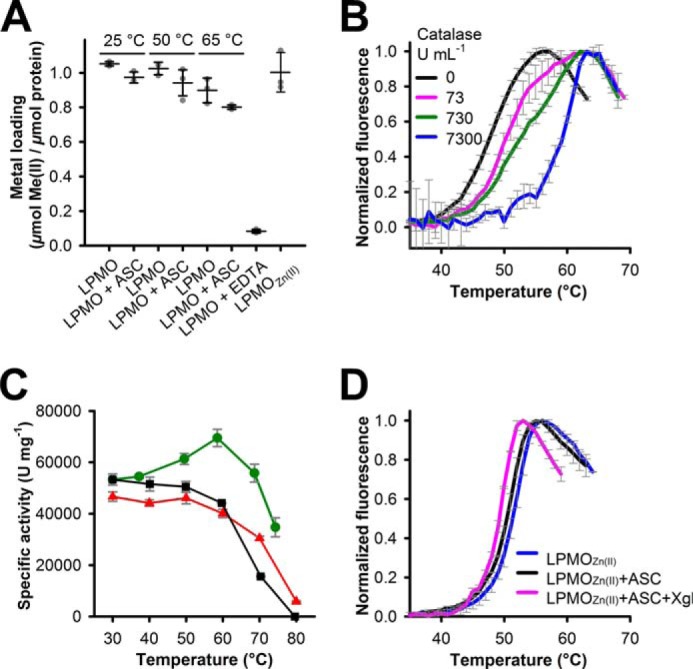
**LPMO unfolding; effect of metal occupancy and effect of catalase.** *A*, copper loading of LPMO (8 μm) in the oxidized and ascorbate-reduced state determined with Zincon (40 μm). Enzymes were incubated for 30 min at the indicated temperatures. The apoenzyme was obtained by incubation of 3 mm enzyme with a 10-fold molar excess of EDTA at 30 °C for 30 min. Apo-LPMO was reconstituted with zinc (LPMO_Zn(II)_) by incubation with a 10-fold molar excess of ZnCl_2_ for 1 h at 30 °C. *B*, unfolding of LPMO (5 μm) in the presence of ascorbic acid and catalase from *C. glutamicum*. Data are expressed as mean values ± S.D. from three independent repeats. *C*, thermal stability of catalase was measured by incubating the enzyme (13 μg ml^−1^) at increasing temperatures for 10 min and measuring residual activity at 30 °C (*black line*, *squares*). Alternatively, catalase activities were determined at increasing temperatures (*green line*, *circles*). Catalase activities (730 units ml^−1^) in the presence of 5 mm ascorbic acid during a heat ramp (30–75 °C; 1 °C min^−1^) were measured for residual catalase activity at 30 °C (*red line*, *triangles*). Data are expressed as mean values ± S.D. from three independent repeats. *D*, fluorescence traces of apo-LPMO (5 μm) reconstituted with ZnCl_2_ measured in the presence of ASC (5 mm) and/or xyloglucan (*Xgl*; 2 mg ml^−1^). Data are expressed as mean values ± S.D. from three independent repeats.

It is well-documented that reducing agents such as ascorbic acid reduce molecular oxygen to H_2_O_2_, which modifies proteins at high concentrations. Also, reactive oxygen species (ROS) are released by LPMO in the absence of a carbohydrate substrate (20, 29). The observed decrease in *T_m_*_,app_ can originate either from the attack of ROS or the dissociation of the copper from the active site. To investigate whether leakage of copper contributes to the unfolding of LPMO, we employed a colorimetric assay to assess the cofactor loading of oxidized and reduced LPMO at different temperatures. For the purified enzyme in its oxidized state, a stoichiometry of 1:1 was determined (Fig. 4*A*), indicating full occupation of all copper-binding sites. Treatment with a 10-fold molar excess of EDTA led to almost complete loss of copper, whereas incubation of LPMO with 10 mm ascorbic acid at 25, 50, and 65 °C for 30 min resulted in a moderate loss of copper. At 65 °C, which is well above the *T_m_*_,app_ of *Nc*LPMO9C, still ∼80% of the copper atoms were bound, which indicates a high affinity of the copper-binding site (Fig. 4*A*). Nevertheless, the addition of the reducing agents ascorbic acid and methoxybenzenediol had severer effects on the stability of LPMO than the copper removal from the active site (Table 1 and Fig. S4 (*A* and *B*)). To test the presence and action of ROS in the unfolding experiments, thermostable catalase from *Corynebacterium glutamicum* (73–7300 units ml^−1^) and superoxide dismutase (0.15 μm) were added to unfolding assays containing LPMO and ascorbic acid. The addition of catalase increased the *T_m_*_,app_ of *Nc*LPMO9C, although high activities were required to fully recover the stability of the LPMO (Table 3 and Fig. 4*B*). The *C. glutamicum* catalase was stable under the tested conditions (Fig. 4*C*). The *T_m_*_,app_ value in the presence of 7300 units ml^−1^ catalase was 61.1 ± 0.8 °C, which is close to the *T_m_*_,app_ of the oxidized enzyme. The addition of superoxide dismutase alone or in combination with catalase had no impact on the *T_m_*_,app_ value of *Nc*LPMO9C or the shape of the fluorescence traces in the presence of ascorbic acid (Table 3 and Fig. S5). Replacement of copper by zinc eliminated the effect of substrate stabilization, and *T_m_*_,app_ values measured in the presence of ascorbic acid and/or xyloglucan were within a range of 3 °C (Fig. 4*D*). These results demonstrate that hydrogen peroxide is produced by LPMO in the thermal stability assays when a reductant and oxygen are present and that the oxidative damage it inflicts on LPMO decreases its stability.

**Table 3:** ***T_m_*_,app_ values of *Nc*LPMO9C in the presence of catalase and superoxide dismutase** *T_m_*_,app_ values were measured in the presence of ANS (200 μm) as outlined under “Experimental procedures.” At a catalase activity of 730 units ml^−1^, two minima of similar intensity were observed in the first derivative of the fluorescence traces. Data are expressed as mean values ± S.D. from three independent repeats. SOD, superoxide dismutase. ND, not detected.

Catalase	SOD (0.15 μm)	*T_m,app_*	*T*_*m*,app 2_
*units ml*^−*1*^		°*C*	°*C*
0	−	48.8 ± 1.1	ND
0	+	49.0 ± 0.3	ND
73	−	49.6 ± 0.5	ND
730	−	50.3 ± 0.5	58.7 ± 1.2
730	+	48.7 ± 1.2	59.0 ± 0.5
7300	−	61.1 ± 0.8	ND

### Copper reduction induces conformational changes in LPMO

**Figure 5.**
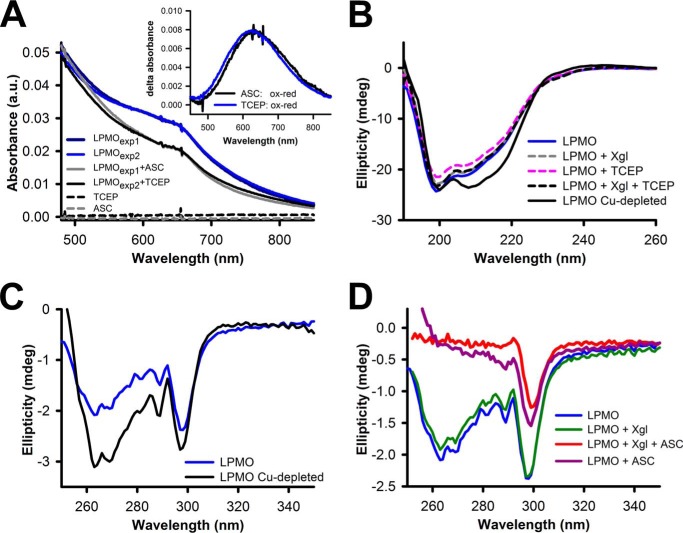
**UV-visible spectra and electronic circular dichroism of *Nc*LPMO9C.** *A*, UV-visible spectra of LPMO (200 μm) in its oxidized state and upon the addition of 2 mm TCEP or 2 mm ascorbate. The *inset* shows differential spectra (oxidized − reduced) for TCEP– and ascorbic acid–reduced LPMO. *B*, ECD spectra in the far-UV range of LPMO (8.8 μm) in its oxidized form or in the presence of the reducing agent TCEP (1 mm) and/or xyloglucan (2 mg ml^−1^). *C*, ECD spectra in the visible range for oxidized LPMO (29 μm) and the apoprotein (29 μm) obtained after incubation with EDTA (see “Experimental procedures” for details). EDTA was removed by extensive rebuffering before conducting the measurements. *D*, effect of ascorbic acid (2 mm) and xyloglucan (2 mg ml^−1^) on the conformation of aromatic amino acids.

Using electronic circular dichroism (ECD), we explored the effect of reduction and substrate binding on the overall protein structure. ECD spectra of LPMO in its oxidized state collected in the far-UV range (190–260 nm) showed an overall negative ellipticity with a minimum at 199 nm (Fig. 5*B*). The spectrum was indicative of a high content of β sheets (∼30%) and a high content of unordered random coils (∼44%). This is in agreement with the protein crystal structure, which shows an overall β-sandwich fold and the presence of extensive loop regions at the flat binding site responsible for substrate recognition (32). In addition, the lengthy and unstructured linker peptide (∼82 amino acids) connecting the catalytic domain to the CBM1 may contribute to the fraction of unordered random coils. To test the effect of copper reduction on the ECD spectra, we employed the strong reducing agent tris(2-carboxyethyl)phosphine (TCEP) because of its low absorbance in the far-UV range. In contrast, ascorbic acid and phenolic reductants have pronounced absorbances in the UV range and are unsuitable for ECD. UV-visible spectra of LPMO showed that the addition of TCEP caused a decrease in absorbance around 620 nm, which accounts for the weak absorbance of the type-2 copper in LPMO (Fig. 5*A*) (29). This absorbance change was identical to the spectral changes induced by ascorbic acid.

TCEP and xyloglucan, either alone or in combination, did not induce any apparent changes in the LPMO secondary structure. ECD spectra of EDTA-treated and copper-depleted LPMO indicated changes in the overall structure as well as in the active site (Fig. 5, *B* and *C*). The addition of xyloglucan to oxidized *Nc*LPMO9C had no effect on the ECD spectrum in the visible range (Fig. 5*D*). However, ascorbic acid induced pronounced spectral changes, which is apparent from the reduction of the negative ellipticity between 250 and 295 nm. A combination of xyloglucan and ascorbic acid did not change the ECD spectrum in the visible range further. These observations could be indicative of a conformational change of the active site upon reduction. Oxidative damage inflicted by the reductant can also contribute to the observed spectra, but it has to be noted that the observed changes in the ECD spectra were also found in the presence of xyloglucan, which protects the enzyme against oxidative damage under the experimental conditions used in this work.

### Copper reduction increases LPMO's affinity to cellulose

**Figure 6.**
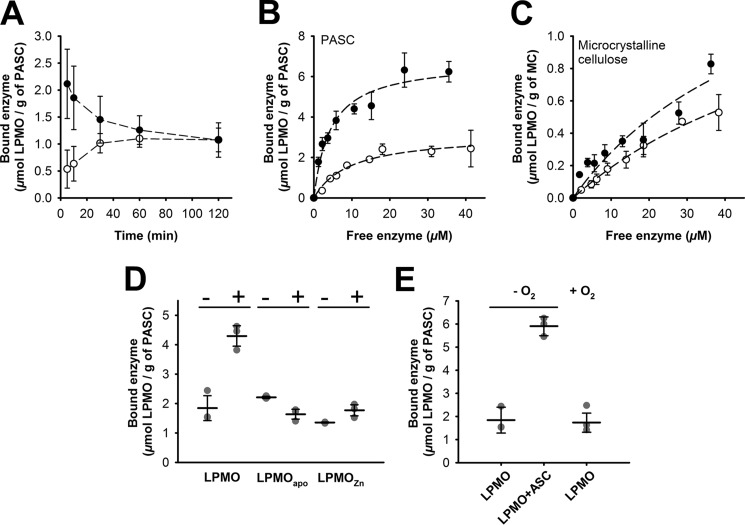
**Binding isotherms and binding assays under atmospheric and anoxic conditions.** *A*, time-dependent binding of oxidized (*empty circles*) and ascorbate-reduced (1 mm ascorbic acid; *filled circles*) LPMO (5 μm) to PASC (1.32 mg ml^−1^). *B*, binding isotherms of reduced (1 mm ascorbic acid; *filled circles*) and oxidized (*empty circles*) LPMO (2–50 μm) to PASC (1.32 mg ml^−1^). Reactions were incubated at 30 °C under constant agitation, and LPMO concentrations in cleared supernatants were measured photometrically (Fig. S6). *C*, binding isotherms for microcrystalline cellulose *(MC*; 10 mg ml^−1^) were measured under the same conditions in the presence (*black circles*) or absence (*empty circles*) of 1 mm ascorbic acid. *D*, binding assays of oxidized LPMO, the apoenzyme, and zinc-loaded LPMO to PASC (1.32 mg ml^−1^) in the absence (−) or presence (+) of 1 mm ascorbic acid. The enzyme concentrations were 24 μm. *E*, the same experiment for native LPMO in the presence and absence of 1 mm ascorbic acid was performed in an anaerobic glove box. *Error bars*, S.D.

The affinity of oxidized and reduced LPMO to amorphous PASC and to microcrystalline cellulose was determined to evaluate the contribution of the copper center to substrate binding. In an initial, time-dependent experiment, 5 μm LPMO was incubated with 1.32 mg ml^−1^ PASC with or without 1 mm ascorbic acid. As is shown in Fig. 6*A*, we observed maximal substrate binding of reduced LPMO within 5 min (the fastest measurement possible), whereas the oxidized enzyme required ∼40 min to reach the binding equilibrium. Over the course of this experiment (2 h), the bound fraction of ascorbic acid–reduced enzyme decreased gradually. This can either be attributed to the depletion of ascorbic acid during the experiment or, more likely, to the modification of the substrate by LPMO activity. In the next step, we quantified the substrate affinity for cellulose by measuring dissociation constants. To avoid excessive substrate degradation and changes of the substrate, incubation times were kept short at 10 min. Dissociation constants for PASC (Fig. 6*B*) revealed a higher affinity of reduced LPMO (*filled circles*; *K_d_* = 4.4 ± 1.0 μm) compared with oxidized LPMO (*empty circles*; *K_d_* = 9.5 ± 2.2 μm). The maximum binding capacity (*B*_max_) for the reduced enzyme was 6.8 ± 0.5 μmol of LPMO/g of PASC, whereas for the oxidized LPMO, a lower value of 3.2 ± 0.3 μmol of LPMO/g of PASC was measured. This translates into partitioning coefficients *K_r_* (*B*_max_/*K_d_*) of 1.55 for reduced and 0.33 for oxidized LPMO. In the case of microcrystalline cellulose (*MC*), saturation could not be achieved, but the fraction of bound enzyme in the presence of a reductant increased notably (Fig. 6*C*). LPMO in its apo-form and LPMO in which the active site copper was replaced by a zinc ion did not show enhanced binding to PASC in the presence of ascorbic acid (Fig. 6*D*). In addition, we performed binding measurements under anaerobic conditions using a 24 μm LPMO concentration and 1.32 mg ml^−1^ of PASC (Fig. 6*E*). In the absence of oxygen and ascorbic acid, no significant change in the amount of the bound LPMO fraction was observed when compared with experiments carried out under atmospheric conditions (Fig. 6*E*). In the presence of ascorbic acid, a 3 times higher amount of LPMO bound to PASC under anaerobic conditions, which confirms that copper reduction alone increased the substrate affinity and that the formation of the activated oxygen species at the copper center did not increase the affinity.

## Discussion

Recent structural and spectroscopic studies revealed important aspects of the reaction mechanism of bacterial and fungal LPMOs (22, 24, 26, 34). Nevertheless, it remains largely elusive how LPMO orchestrates its complex reaction mechanism with substrate binding and how substrates and cosubstrates influence enzyme stability.

Analyses of *Nc*LPMO9C in its oxidized state showed the highest thermal stability in the pH range from pH 6 to 8. The observed stability maximum at pH 6.0 coincides with the pH optimum of the measured copper-reduction rates of *Nc*LPMO9C by its assumed natural interaction partner, cellobiose dehydrogenase (17). At pH values <5, the *T_m_*_,app_ dropped by >10 °C, pointing toward destabilizing protonation effects. Recent crystallographic studies of a fungal LPMO from *Lentinus similis*, whose structure was resolved at pH 5.5 (24) and pH 3.5 (35), showed a distortion of the active-site geometry at low pH, which indicates protein destabilization. We observed the lowest *T_m_*_,app_ in citrate buffer, which was also used for crystallization of *L. similis* LPMO at low pH. Citrate has chelating properties and could interact with the active-site copper.

Because reduction of the active-site copper initiates LPMO activity, we also studied the impact of copper reduction on enzyme stability. The presence of reductants had a strong negative effect on protein stability, demonstrated by an earlier onset of fluorescence traces and broader transition ranges when compared with the oxidized enzyme. This indicates a continuous degradation process occurring during the unfolding experiment, which leads to an additional destabilization of the protein fold. Two possibilities for the destabilization effect were investigated: (i) the loss of the copper atom from the active site and (ii) the formation of amino acid–modifying ROS. ROS can be generated via an uncoupling reaction at the reduced copper center or from the oxidation of the reductant.

The removal of copper from the LPMO active site by EDTA reduced its *T_m_*_,app_ from 61.5 to 52.7 °C, which demonstrates the importance of the histidine brace for the stabilization of the protein fold. Loss of thermal stability due to copper removal has also been reported for a bacterial LPMO from *B. amyloliquefaciens* (30). The replacement of the copper ion by a zinc ion in the active site of *Nc*LPMO9C resulted in a *T_m_*_,app_ of 53 °C, which is similar to the *T_m_*_,app_ of the apoprotein. For the previously investigated *B. amyloliquefaciens* LPMO, a stronger stabilization by zinc was found (30), indicating a higher affinity of this LPMO for zinc. The *K_d_* value of fungal and bacterial LPMOs for Cu^2+^ typically is in the low nanomolar range, whereas other metal ions did not show a comparable binding or activity (5, 30, 36). In a bacterial LPMO, it was shown that Cu^+^ binds much stronger than Cu^2+^ (1.2 nm *versus* 55 nm (11)), which indicates that the dissociation of the active-site copper upon reduction may not be the primary reason for the observed reduction of the *T_m_*_,app_ in the unfolding assays. Based on our findings, ∼80% of the LPMO molecules still contained a copper atom after unfolding experiments under reducing conditions. This excludes the loss of copper from the active site as the initial and main factor for the observed destabilization of LPMO in the presence of reductants.

Next, the effect of ROS generated in the experiment in the presence of reductants was investigated. The addition of the H_2_O_2_ scavenger catalase sufficed to fully stabilize *Nc*LPMO9C under reducing conditions, whereas the addition of superoxide dismutase showed no effect. We therefore conclude that H_2_O_2_ is the destabilizing ROS. These findings are consistent with a recent study showing that severe autoxidation in the vicinity of LPMO's active site occurred in the presence of ascorbic acid and in the absence of a suitable substrate (22). In particular, severe oxidation of the two conserved copper-coordinating histidine residues was observed, which was proposed to be a main factor for enzyme deactivation. In agreement with these results, we found that the presence of carbohydrate substrates recovered the stability and increased the *T_m_*_,app_ of *Nc*LPMO9C in the presence of reductants to the *T_m_*_,app_ of the oxidized enzyme. This was not observed for non-β-glucan polysaccharides or cellobiose. In addition to the previously reported substrate-induced suppression of H_2_O_2_ production by LPMO (14, 29), we suggest the fluorimetric assay used here as a simple and easy-to-apply screening method for the substrate specificity of LPMOs. In a similar approach based on differential scanning fluorimetry, the *T_m_*_,app_ of the copper-saturated bacterial LPMO from *B. amyloliquefaciens* increased by 3.5 °C in the presence of substrate (chitin) (37). This effect was even higher when the LPMO was incubated with chitin in the absence of copper (Δ*T* = 8.3 °C), an effect not observed for *Nc*LPMO9C. These experiments, however, did not consider the reduction state of the enzyme. The presence of substrate most likely reduces deleterious uncoupling reactions of the reduced copper and therefore limits the generation of H_2_O_2_. According to the catalytic mechanism recently reported by Bissaro *et al.* (22), hydrogen peroxide can be used as cosubstrate by LPMO, which would then deplete the formed H_2_O_2_ in experiments containing a carbohydrate substrate.

Following the copper reduction by electronic circular dichroism, we observed conformational changes in LPMO. It is noteworthy that small conformational changes in the copper center of a bacterial LPMO upon metal binding have also been observed in a previous NMR study (11). In the case of *Nc*LPMO9C, the observed conformational changes inflicted by reduction were more pronounced than those observed for copper removal. Using EPR spectroscopy, Borisova *et al.* (32) first demonstrated that substrate binding induced conformational changes in the active site of *Nc*LPMO9C. In the substrate-bound crystal structure of *L. similis* LPMO at pH 5.5, the coordination sphere of the reduced copper notably changed upon substrate binding (24). In this enzyme-substrate complex, oxidized LPMO was found in coordination with a chloride ion, which may be a substitute for an activated oxygen intermediate. A similar effect has also been shown for *Nc*LPMO9C; the addition of cyanide, which tightly binds copper and mimics an activated oxygen species, enhances the substrate binding of the enzyme (26). Our results extend these observations and provide direct evidence that the reduction of the LPMO active-site copper is a driving force for substrate binding. The measured dissociation constants revealed a 2-fold higher affinity of LPMO for cellulose in the presence of a reducing agent. The partitioning coefficient for reduced LPMO was 4.7-fold lower than for oxidized LPMO. Binding assays of reduced LPMO in an oxygen-free environment, and hence in the absence of a cosubstrate (either O_2_ or H_2_O_2_), demonstrated that copper reduction alone is sufficient to promote substrate binding. Therefore, a scenario in which the reduced LPMO initially binds to cellulose before it activates the cosubstrate is also feasible.

After completion of the catalytic cycle, reoxidized LPMO with its lower affinity for cellulose could dissociate more easily from the substrate to become available for reduction by cellobiose dehydrogenase, after which the next catalytic turnover can be initiated. If not oxygen but instead H_2_O_2_ is present and used, LPMO would remain in the reduced state after a catalytic cycle (22), and the probability of dissociating from the polymeric substrate would be lower.

In both scenarios, the reactive oxygen intermediate is formed only when the preliminarily reduced active site is in intimate contact with the polysaccharide. Such a mechanism could effectively prevent a deleterious uncoupling reaction that generates H_2_O_2_ or the attack of the reactive oxygen intermediate on vicinal amino acids. In this respect, it is interesting to note that many fungal LPMOs possess CBM1. In our case, the CBM1 did not impact the thermal stability of the enzyme. Deactivation of the active site by EDTA resulted in a substantial loss of stability, regardless of the presence of cellulose or xyloglucan. Crouch *et al.* (38) previously tested the binding of two cellulose-active LPMOs with and without CBMs and showed that CBMs can in fact modulate the reactivity of activated LPMOs. In particular, the ratio between oxidized and non-oxidized reaction products was changed. Along these lines, the presence of a CBM appears to be beneficial in some cases but not essential for the function of LPMOs. If present, the CBM may ensure a basic affinity of LPMO at any time during catalysis, thus keeping LPMO close to the substrate surface.

In this work, we measured the oxidative damage-induced unfolding of LPMO9C from *N. crassa*. This was not observed in the absence of the active-site copper or in the absence of a reducing agent. Most notably, oxidative damage could be prevented by the addition of suitable substrates. We conclude that the reduced active-site copper is essential for substrate recognition, which prevents the generation and accumulation of reactive oxygen species and protects the enzyme during catalysis.

## Experimental procedures

### Materials and chemicals

Buffer salts, 1,2,3-trihydroxybenzene (pyrogallol), 3,4,5-trihydroxybenzoic acid (gallic acid), ascorbic acid, TCEP, methoxyhydroquinone, EDTA, 2-carboxy-2′-hydroxy-5′-sulfoformazylbenzene (Zincon), ANS, dextran, CMC, and maltodextrin (dextrose-equivalent 13–17) were purchased from Sigma-Aldrich. Microcrystalline cellulose (20–160 μm) was from Merck, and birchwood xylan (purity >90%) was from Roth. Xyloglucan from *Tamarindus indicus* (purity ∼95%) and arabinogalactan from larch (purity ∼95%; Ara/Gal = 15:85) were purchased from Megazyme. PASC was prepared by dissolving 4 g of microcrystalline cellulose in 100 ml of ice-cold 85% (by weight) phosphoric acid. The solubilized cellulose was stirred for 1 h at 4 °C and precipitated by the addition of 1,900 ml of ice-cold HQ-water. The cellulose was washed on a vacuum filtration system with ∼2 liters of ice-cold water, 2 liters of a 2 m sodium bicarbonate solution, and 1 liter of 50 mm sodium phosphate buffer, pH 6. Finally, PASC was homogenized with a high-performance disperser (Ultra Turrax, Ika).

### Enzymes

The lytic polysaccharide monooxygenase LPMO9C from the ascomycete *N. crassa* was recombinantly expressed in *Pichia pastoris* X-33 as reported previously (39). Production in a 5-liter laboratory fermenter and column purification were done according to a published protocol (29). The purity of the enzyme was checked by SDS-PAGE. Superoxide dismutase from bovine erythrocytes, peroxidase from horseradish, and catalase from *C. glutamicum* were purchased from Sigma-Aldrich. Activity of catalase was determined by measuring the decrease of H_2_O_2_ at 240 nm (ϵ_240_ = 46,900 mm^−1^ cm^−1^) (40) in 100 mm sodium phosphate buffer, pH 6.0. Reactions were monitored in an Agilent 8453 UV-visible spectrometer featuring a photodiode array detector and a temperature-controlled 8-cell changer. One unit of catalase activity is defined as the amount of enzyme consuming 1 μmol of H_2_O_2_/min under the specified assay conditions. Blank runs in the absence of catalase were recorded for all conditions and subtracted from the catalase reactions. Catalase concentrations were determined with the Bradford protein assay (Bio-Rad) using BSA as a calibration standard.

### Stability measurements

Thermal unfolding of *Nc*LPMO9C was recorded on a Cary Eclipse fluorescence spectrophotometer equipped with a temperature-controlled 4-cell holder and two cuvette-based temperature probes (all equipment from Agilent Technologies). In these experiments, a temperature ramp from 30 to 75 °C was applied with increments of 1 °C/min. Fluorescence scans of *Nc*LPMO9C (10 μm) showed the highest changes in the fluorescence signal when using an excitation wavelength of 279 nm and an emission wavelength of 349 nm. Due to the small overall changes upon unfolding, unfolding processes were routinely monitored in the presence of ANS, which yields high fluorescence signals when binding to (partly) unfolded proteins. In this setup, the excitation wavelength was set to 378 nm, whereas the highest emission of ANS-protein complexes was observed at 480 nm. Reactions were carried out in stirred quartz cuvettes (path length 10 mm) in a total volume of 2 ml. The internal temperature probes allowed accurate control over the reaction conditions. Initially, buffer solutions containing ANS (200 μm), polysaccharide substrates, and/or EDTA were pre-equilibrated to 30 °C for ∼5 min. For measurements of LPMO in the reduced state, reductants were added to the cuvettes ∼1 min before starting the unfolding assays to minimize possible side reactions. Reactions were initiated by the addition of 5 μm *Nc*LPMO9C from a concentrated stock solution. All reductants were freshly prepared for each unfolding experiment. TCEP was not included as a reductant for stability measurements, because LPMO precipitated rapidly in its presence above ∼50 °C. All measurements were corrected for baseline fluorescence recorded in the absence of enzyme. Buffer systems used were 50 mm sodium acetate (pH 4–5.6), 50 mm sodium citrate (pH 4–6), 50 mm MOPS (pH 6–8), and 50 mm potassium phosphate (pH 6–8). *T_m_*_,app_ values were calculated from the first derivative of the fluorescence traces, in which the negative peak indicates the *T_m_*_,app_ value.

### LPMO activity based on hydrogen peroxide release

The futile oxygen-reducing activity of LPMO in the absence of substrate was measured with the Amplex Red/horseradish peroxidase assay according to a published protocol (29). Reactions had a total volume of 200 μl and were measured in black polystyrene 96-well plates using a PerkinElmer Life Sciences EnSpire Multimode plate reader at room temperature (23 °C). To initiate the reaction, 180 μl of a reaction mix containing 55.5 μm Amplex Red and 7.92 units ml^−1^ horseradish peroxidase were mixed with 20 μl of the sample solutions containing 0.3–0.5 mg ml^−1^ LPMO. Measurements were performed in 50 mm sodium phosphate buffer, pH 6, using an excitation wavelength of 569 nm and an emission wavelength of 585 nm. Activities were determined from six independent repeats ± S.D.

### ECD spectroscopy

ECD spectra were collected on a Chirascan circular dichroism spectrometer (all equipment from Applied Photophysics). The instrument was flushed with a nitrogen flow of 5 liter min^−1^ throughout all experiments, and the temperature was kept constant at 25 °C. *Nc*LPMO9C was analyzed in the far-UV region (190–260 nm) at a scan speed of 5 s nm^−1^ and at a bandwidth of 1 nm. Cuvettes had a path length of 1 mm and were loaded with a protein concentration of 0.3 mg ml^−1^. Measurements in the near-UV and visible region (260–350 nm) were carried out at a protein concentration of 1 mg ml^−1^ in cuvettes with a path length of 10 mm. All measurements were performed in 50 mm phosphate buffer, pH 6. Data were analyzed with the PRO-DATA SX software version 2.2.17 (Applied Photophysics).

### Copper loading

LPMO concentrations were determined photometrically at 280 nm in 50 mm borate buffer, pH 9, containing 8 m urea. A molar absorption coefficient of 46.91 mm^−1^ cm^−1^ at 280 nm (ϵ_280_) was calculated from the protein sequence using the ProtParam online tool (http://web.expasy.org/protparam/). The copper content of *Nc*LPMO9C was determined colorimetrically using the metal chelator Zincon (41). Briefly, 8 μm LPMO was added to 50 mm borate buffer, pH 9, containing 8 m urea and 40 μm Zincon. After incubation for 10 min at 23 °C, the absorbance at 610 nm was recorded on a diode array spectrophotometer and compared with a calibration curve generated with copper chloride. Samples incubated with ascorbic acid underwent extensive buffer exchange using centrifugal concentration tubes with a polyethersulfone membrane (cut-off 10 kDa; 20,238 × *g* for 2 min) to remove ascorbic acid and unbound copper. At each rebuffering step, the added buffer was brought to the respective incubation temperature (25, 50, or 65 °C).

The apo-form of LPMO was generated by adding 30 mm EDTA to 3 mm LPMO in 100 mm sodium acetate buffer, pH 5.6 (final concentrations). The mixture (100 μl) was incubated for 30 min at 30 °C before removing EDTA by extensive rebuffering using centrifugation tubes. Zn^2+^-loaded LPMO was obtained by incubation of apo-LPMO (∼1 mm) with 10 mm ZnCl_2_ for 30 min at 30 °C. The excess of ZnCl_2_ was removed using centrifugation tubes with a 10-kDa cut-off.

### Quantification of ascorbic acid

Ascorbic acid concentrations were determined via oxidation of dichloroindophenol. The stoichiometry of the reaction is 1:1. Sample solutions containing ascorbic acid were mixed with a solution of 300 μm dichloroindophenol in 50 mm phosphate buffer, pH 6.0. Concentrations of ascorbic acid were calculated from the decrease in dichloroindophenol absorbance at 520 nm (ϵ_520_ = 6.9 mm cm^−1^). Absorbances were recorded on a diode array spectrophotometer at room temperature (23 °C). All data points are expressed as the mean ± S.D. of three independent repeats.

### Binding of LPMO to cellulose

Binding of oxidized and reduced LPMO to insoluble cellulose was measured at 30 °C in 50 mm phosphate buffer, pH 6. Reactions contained microcrystalline cellulose (10 mg ml^−1^) or PASC (1.32 mg ml^−1^) and 2–50 μm *Nc*LPMO9C in a final volume of 500 μl. Reaction mixtures were incubated under constant agitation (800 rpm) for 10 min to avoid excessive degradation of the substrates. Cellulose with bound LPMO was removed by centrifugation (20,238 × *g* for 3 min), and the concentration of free enzyme in the supernatant was measured photometrically at 280 nm on an Agilent 8453 UV-visible spectrophotometer (Agilent Technologies). Isotherms for reduced LPMO were measured in the presence of 1 mm ascorbic acid. In these experiments, the dominant absorbance of ascorbic acid in the UV range was quenched by the addition of phosphoric acid, which was added from an 85% (by weight) stock solution to a final concentration of 240 mm. This resulted in a hypsochromic shift of the ascorbic acid absorption, which allowed photometric determination of the protein concentration (Fig. S6). LPMO concentrations in phosphoric acid–treated samples were determined at 290 nm, where no interference with residual absorbance of ascorbic acid was observed. All data points are expressed as the mean ± S.D. of three independent repeats and were fitted to a one-site binding model using Sigma Plot version 12.5 (Systat Software, Inc.).

Binding of LPMO to cellulose in the absence of oxygen was studied in a glove box (Whitley DG250, Don Whitley Scientific, Shipley, UK) which was continuously flushed with a nitrogen/hydrogen mixture (99:1). Residual oxygen was removed with a built-in palladium catalyst, and the generated water vapor was absorbed by silica gel. Electronic absorption spectra were recorded with an Agilent 8453 UV-visible spectrophotometer equipped with a photodiode array detector. All reagents and enzyme solutions used in the glove box were extensively degassed by applying alternate cycles of vacuum and nitrogen pressure. Experiments were carried out in stirred quartz cuvettes at room temperature (23 °C). Otherwise, the experimental setup was identical to the measurements carried out at ambient conditions. Experiments were done in three independent repeats (mean ± S.D.).

## Author contributions

D.K. and R.L. conceptualization; D.K., P.G.F., and R.L. data curation; D.K., P.G.F., and R.L. supervision; D.K., M.A., P.G.F., and R.L. investigation; D.K. and M.A. visualization; D.K. and R.L. methodology; D.K. and R.L. writing-original draft; D.K. and R.L. writing-review and editing; P.G.F. and R.L. resources; R.L. funding acquisition.

## Supplementary Material

Supporting Information

## Footnotes

4 The abbreviations used are:

LPMO: lytic polysaccharide monooxygenase
ANS: 8-anilinonaphthalene-1-sulfonic acid
TCEP: tris(2-carboxyethyl)phosphine
ECD: electronic circular dichroism
*T_m_*_,app_: apparent transition midpoint temperature
CBM: carbohydrate-binding module
ASC: ascorbic acid
CMC: carboxymethylcellulose
PASC: phosphoric acid-swollen cellulose
ROS: reactive oxygen species.
