# Supplementary material for: Active-site copper reduction promotes substrate binding of fungal lytic polysaccharide monooxygenase and reduces stability
Source: J Biol Chem. 2017 Dec 19;293(5):1676–87. doi: 10.1074/jbc.RA117.000109 (PMC5798298; doi:10.1074/jbc.RA117.000109)
Supplement: Supporting Information [file supp_293_5_1676__index.html]

Active-site copper reduction promotes substrate binding of fungal lytic polysaccharide monooxygenase and reduces stability — Stability and substrate binding of LPMO — Active-site copper reduction promotes substrate binding of fungal lytic polysaccharide monooxygenase and reduces stability — Stability and substrate binding of LPMO — Supporting Information 

# Active-site copper reduction promotes substrate binding of fungal lytic polysaccharide monooxygenase and reduces stability

## Supporting Information

- Active-site copper reduction promotes substrate binding of fungal lytic polysaccharide monooxygenase and reduces stability - Supporting Information
