# Supplementary material for: Active-site copper reduction promotes substrate binding of fungal lytic polysaccharide monooxygenase and reduces stability
Source: J Biol Chem. 2017 Dec 19;293(5):1676–87. doi: 10.1074/jbc.RA117.000109 (PMC5798298; doi:10.1074/jbc.RA117.000109)
Supplement: Supporting Information [file supp_RA117.000109_132646_1_supp_37375_p0qq2n.pdf]

Supporting Information

Active-site copper reduction promotes substrate binding of fungal lytic polysaccharide monooxygenase and reduces stability

Daniel Kracher[1], Martina Andlar[1], Paul G. Furtmüller[2], and Roland Ludwig[1]

[1] Biocatalysis and Biosensing Research Group, Department of Food Science and Technology, BOKU - University of Natural Resources and Life Sciences, Muthgasse 18, 1190 Vienna, Austria

[2] Division of Biochemistry, Department of Chemistry, BOKU - University of Natural Resources and Life Sciences, Muthgasse 18, 1190 Vienna, Austria

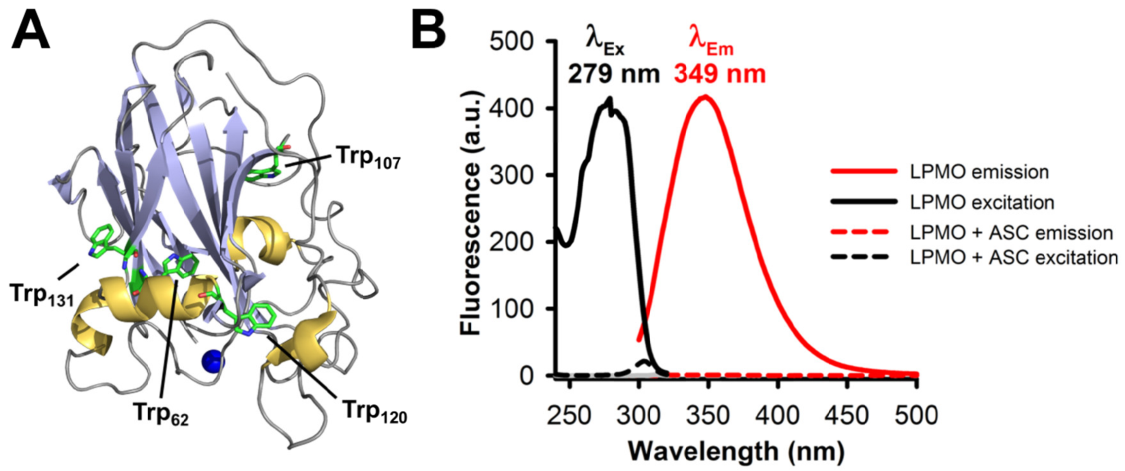

Figure S1. **Molecular structure and fluorescence spectra of *Nc*LPMO9C**. *A*, ribbon drawing of *Nc*LPMO9C (pdb: 4D7U) with indicated tryptophan residues. The active-site copper is shown as blue sphere. *B*, Excitation and emission spectra of *Nc*LPMO9C (10 μM) in its oxidized state (solid lines) and after addition of ascorbic acid to a final concentration of 5 mM (dashed lines). Spectra were recorded at 30 °C in 50 mM sodium phosphate buffer, pH 6.0, at a scan speed of 10 nm s^{-1}.

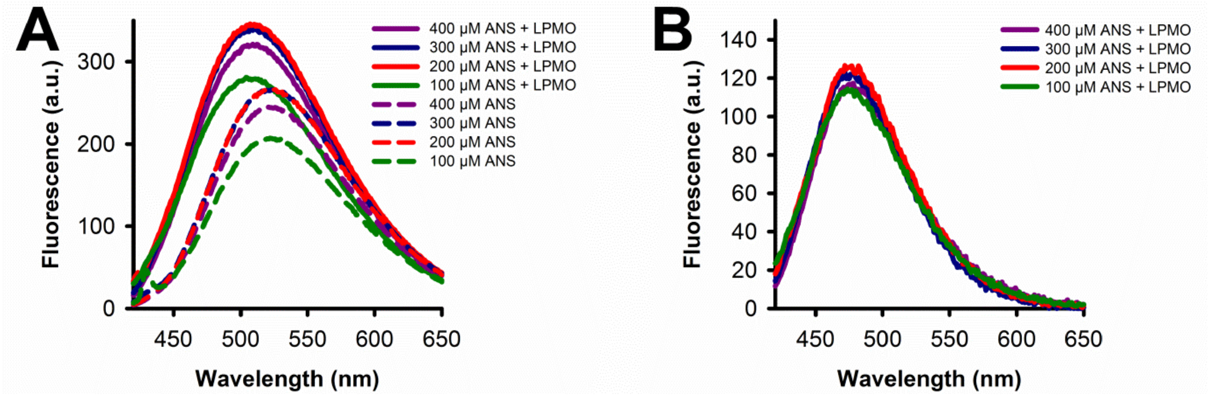

Figure S2. Emission spectra of ANS-LPMO complexes. *A*, fluorescence spectra of oxidized LPMO recorded at 63 °C, where highest fluorescence emission intensity was observed. Experiments were carried out in 50 mM phosphate buffer, pH 6.0. Highest fluorescence intensities were observed using 200 μM ANS, which is a 40-fold molar excess over *Nc*LPMO9C. At higher ANS concentrations, fluorescence intensities decreased slightly. *B*, differential spectra obtained by subtraction of ANS spectra from spectra of LPMO-ANS complexes. Maximal emission at 480 nm was observed upon excitation at 378 nm.

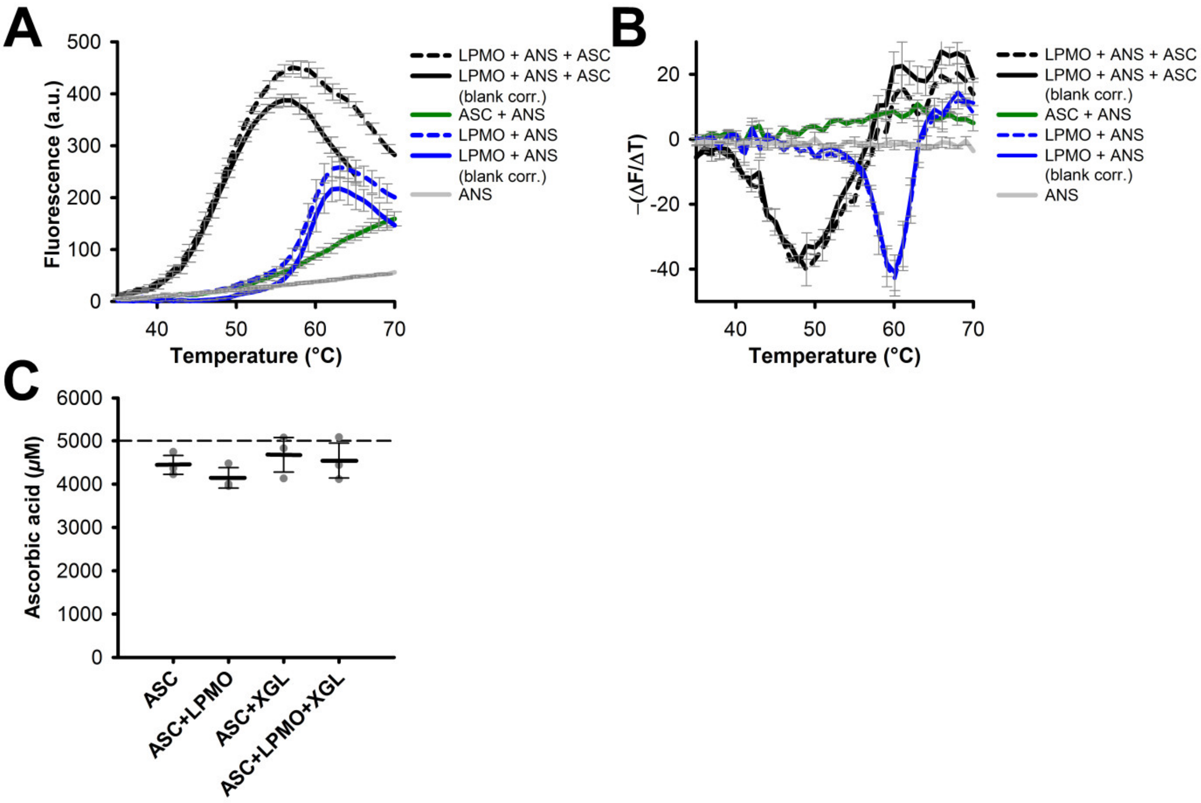

Figure S3. Unfolding of *Nc*LPMO9C (5 μM) in presence of ANS (200 μM) and ascorbic acid (ASC, 5 mM). *A*, raw traces showing fluorescence emission intensities for LPMO/ANS complexes in presence or absence of ascorbic acid. For blank-corrected traces, fluorescence of ANS/ascorbic acid or ANS was subtracted. *B*, first derivative of unfolding traces shown in *A*. Data from unfolding experiments are expressed as mean values (± SD) from three independent repeats. *C*, remaining ascorbic acid concentrations measured after completion of unfolding assays (30 °C – 75 °C at a rate of 1 °C min^{-1}). Ascorbic acid was measured by adding 20 μL of the sample solution to a solution of 300 μM 2,6-dichloroindophenol. The initial concentration of ascorbic acid was 5 mM (dashed line). Absorbance of 2,6-dichloroindophenol was recorded at 520 nm ($\varepsilon_{520} = 6.9$ mM cm^{-1}). Data are expressed as mean values (± SD) from three independent repeats.

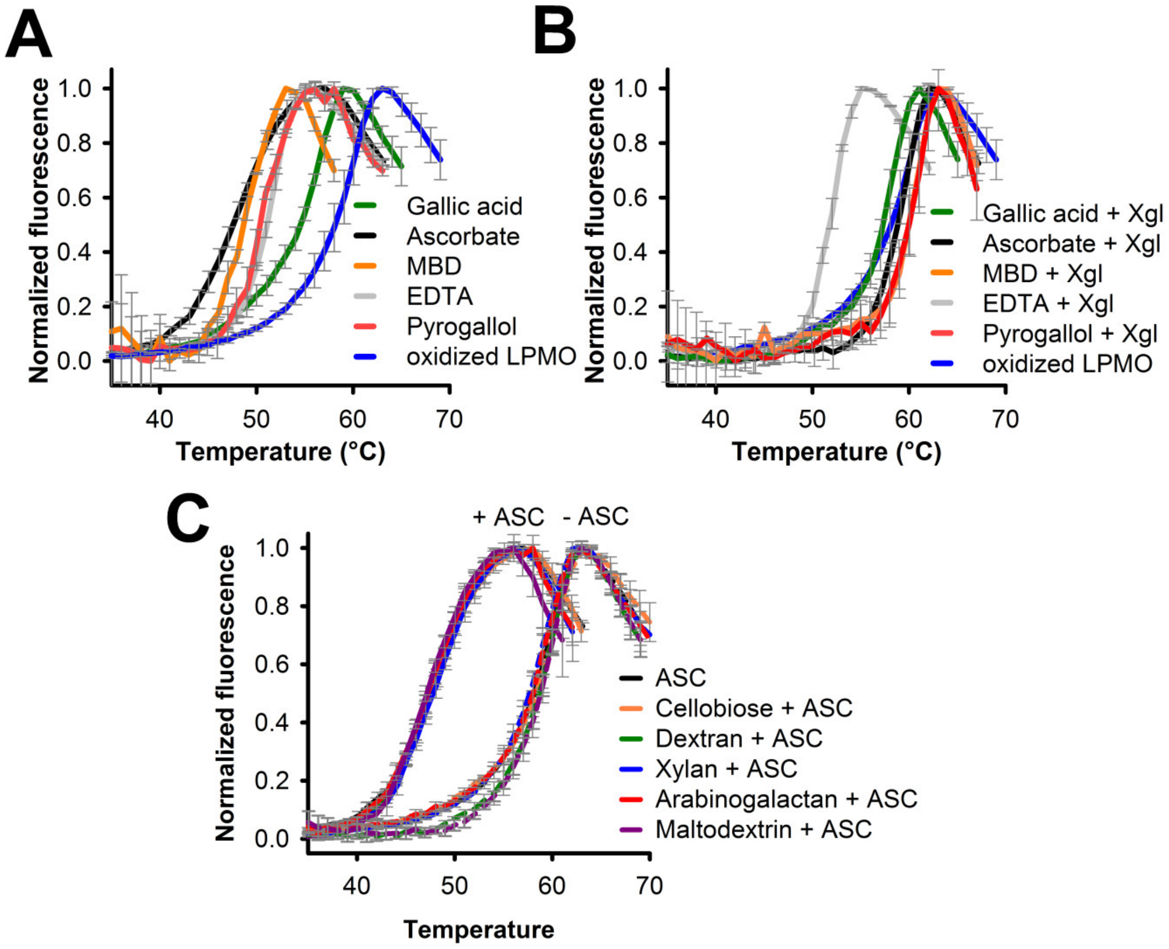

Figure S4. Effect of reductants, unspecific carbohydrates and EDTA on the thermostability of LPMO. *A*, fluorescence emission traces of *Nc*LPMO9C recorded in presence of various reducing agents (2 mM, final concentration) and EDTA (0.3 mM, final concentration); MB, methoxybenzenediol. *B*, fluorescence emission traces of *Nc*LPMO9C in presence of 2 mM reducing agents or 0.3 mM EDTA and 2 mg mL^{-1} xyloglucan. *C*, Unfolding emission traces of ascorbic acid-reduced (+ASC) and oxidized (-ASC) LPMO upon addition of unspecific carbohydrates. Error bars denote the standard deviation (± SD) from three independent repeats.

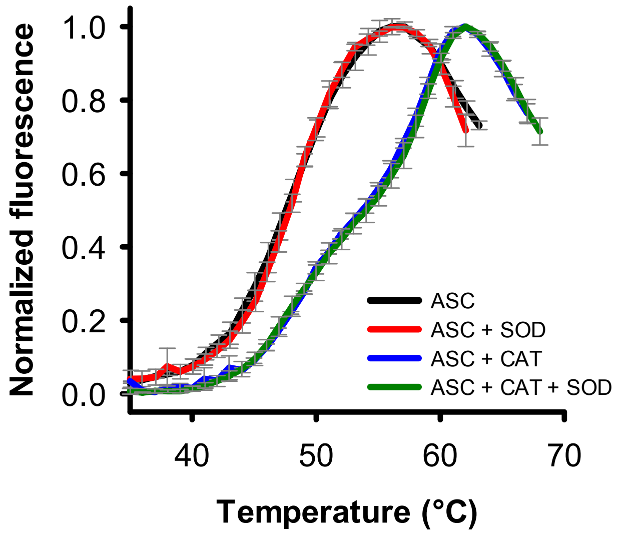

Figure S5. Effect of ascorbic acid (ASC, 5 mM), superoxide dismutase (SOD, 0.15 *μ*M) and catalase (CAT, 720 U mL^{-1}) on the unfolding of LPMO.

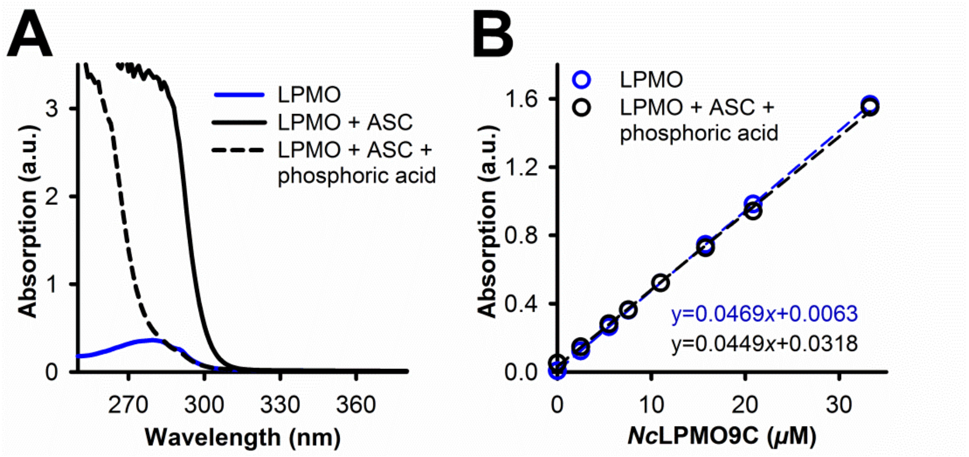

Figure S6. Photometric determination of *Nc*LPMO9C in presence of ascorbic acid (ASC). *A*, UV-visible spectra of *Nc*LPMO9C (5 *μ*M) in 50 mM sodium phosphate buffer, pH 6.0 (blue line) and upon addition of 1 mM ascorbic acid (black line). Spectra were recorded at room temperature (~23 °C) using a diode array spectrophotometer. Absorbance of ascorbic acid shifted in presence of 240 mM phosphoric acid (dashed line). *B*, Absorbance of increasing *Nc*LPMO9C concentrations in 50 mM phosphate buffer, pH 6.0 (blue circles). To the same samples 1 mM ascorbic acid and 240 mM phosphoric acid were added (black circles). Color-coded dashed lines indicate the linear fit.
